# Emergence of Mechano-Sensitive Contraction Autoregulation in Cardiomyocytes

**DOI:** 10.3390/life11060503

**Published:** 2021-05-29

**Authors:** Leighton Izu, Rafael Shimkunas, Zhong Jian, Bence Hegyi, Mohammad Kazemi-Lari, Anthony Baker, John Shaw, Tamas Banyasz, Ye Chen-Izu

**Affiliations:** 1 Department of Pharmacology, University of California, Davis, CA 95616, USA; rshimkunas@gmail.com (R.S.); zjian@ucdavis.edu (Z.J.); bhegyi@ucdavis.edu (B.H.); mkazemilari@ucdavis.edu (M.K.-L.); banyasz.tamas@med.unideb.hu (T.B.); ychenizu@ucdavis.edu (Y.C.-I.); 2 Department of Medicine, University of California, San Francisco, CA 94121, USA; Anthony.Baker@ucsf.edu; 3 Department of Aerospace Engineering, University of Michigan, Ann Arbor, MI 48109, USA; jashaw@umich.edu; 4 Department of Physiology, University of Debrecen, 4032 Debrecen, Hungary; 5 Department of Biomedical Engineering, University of California, Davis, CA 95616, USA; 6 Department of Internal Medicine, Division of Cardiology, University of California, Davis, CA 95616, USA

**Keywords:** autoregulation, contractility, cardiomyocyte, Anrep effect, mathematical analysis, mathematical model, mechano-chemo-transduction

## Abstract

The heart has two intrinsic mechanisms to enhance contractile strength that compensate for increased mechanical load to help maintain cardiac output. When vascular resistance increases the ventricular chamber initially expands causing an immediate length-dependent increase of contraction force via the Frank-Starling mechanism. Additionally, the stress-dependent Anrep effect slowly increases contraction force that results in the recovery of the chamber volume towards its initial state. The Anrep effect poses a paradox: how can the cardiomyocyte maintain higher contractility even after the cell length has recovered its initial length? Here we propose a surface mechanosensor model that enables the cardiomyocyte to sense different mechanical stresses at the same mechanical strain. The cell-surface mechanosensor is coupled to a mechano-chemo-transduction feedback mechanism involving three elements: surface mechanosensor strain, intracellular ${\mathrm{Ca}}^{2+}$ transient, and cell strain. We show that in this simple yet general system, contractility autoregulation naturally emerges, enabling the cardiomyocyte to maintain contraction amplitude despite changes in a range of afterloads. These nontrivial model predictions have been experimentally confirmed. Hence, this model provides a new conceptual framework for understanding the contractility autoregulation in cardiomyocytes, which contributes to the heart’s intrinsic adaptivity to mechanical load changes in health and diseases.

## 1. Introduction

**Figure 1:**
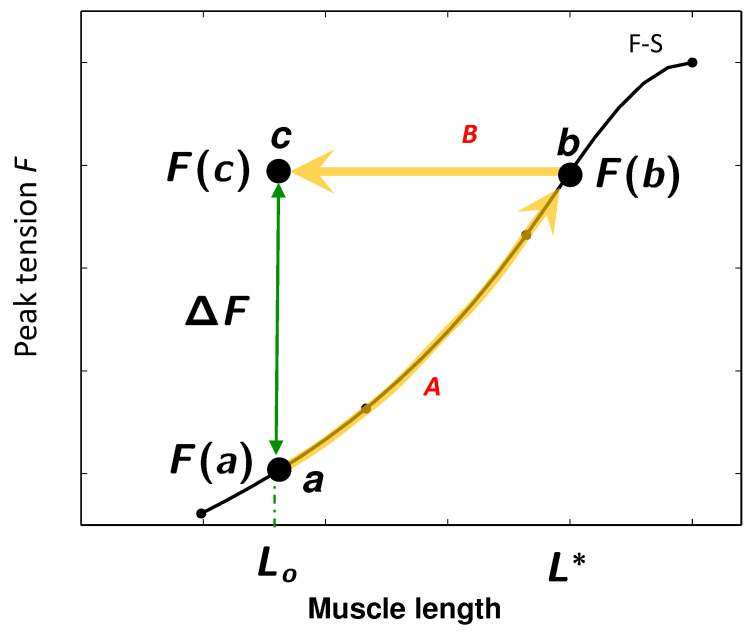
Load-adaptation property of cardiac muscle. Black curve shows the relationship between length of a fiber in the heart and force generated by Frank-Starling (F-S) mechanism. Sudden increase of outflow resistance causes an initial diastolic fiber length increase from $L_{o}$ to $L^{*}$ and concomitant contractile force increase from F(a) to F(b) by the F-S mechanism. For the experiments of Cingolani et al. [5] the chamber volume returns to the original volume (muscle length $L_{0}$) but the contractile force must be F(c)=F(b). The adaptive force ΔF (green double-headed arrow) is the force needed to account for the Anrep effect. F-S curve redrawn from Allen and Kurihara [2].

Ernest H. Starling found through a series of brilliant experiments that “the mechanical energy set free on passage from the resting to the contracted state of contraction depends on … the length of the muscle fiber [1] p. 472.” He recognized that this relationship was so important that he called it the “law of the heart”. Implicit in this law is that the force of contraction is a single-valued function of the muscle length as shown in Figure 1 adapted from Allen and Kurihara’s experiment on trabeculae [2]. Starling’s law of the heart is a foundation stone of cardiac physiology and it underpins our understanding of heart function in both normal and diseased states [3]. But even as Starling was doing his experiments, Gleb von Anrep’s experiments showed that muscle fiber length was not the sole determinant of contractile force. Anrep found that increasing the arterial resistance caused an initial increase in the diastolic and systolic volumes of the heart but after a few minutes the heart returned to near its initial volumes indicating that the heart was able to maintain an increased level of contraction force at a smaller fiber length despite the high arterial resistance [4].

Work by Rosenblueth et al. [6], Clancy et al. [7], and Klautz et al. [8] have confirmed Anrep’s finding that upon an increase of vascular resistance, the end-diastolic volume (EDV, a measure of relaxed muscle fiber length) shows an initial transient increase that gradually diminishes despite the continued higher outflow resistance. Indeed, Sarnoff et al. [9] in 1960 and O. Cingolani et al. [5] in 2011 have shown that the EDV can return the very same value the heart had before the outflow resistance increased.

We have illustrated the trajectory of the response of the heart to an increase of outflow resistance on Figure 1, associating fiber length *L* with left ventricular (LV) volume at diastole and fiber tension *F* at systole with peak LV pressure. Suppose that before the outflow resistance is increased the fiber length at end-diastole is $L_{o}$. When the outflow resistance is increased there is an initial increase in the end-diastolic fiber length to $L^{*}$ (path *A* marked by the heavy yellow arrow). The increase in fiber length results in a greater contraction force F(b) as indicated on Allen & Kurihara’s Frank-Starling (F-S) curve. This force F(b) is needed to overcome the increased outflow resistance.

As Anrep and others observed end-diastolic LV volume decreases over time even though the outflow resistance remained elevated. The trajectory of the EDV during this phase is along path *B* in Figure 1. This path is horizontal because the larger contraction force F(b) must be maintained to eject blood against the maintained higher outflow resistance. When the heart reaches a steady state (ss) the fiber length is $L_{ss}$. In Sarnoff et al.’s and O. Cingolani et al.’s experiments the steady-state fiber length equals the original length, $L_{ss}=L_{o}$. Because the outflow resistance remains high, the force of contraction at the steady state must be F(c)=F(b).

Regardless of the value of $L_{ss}$, it is clear from Figure 1 that cardiac muscle is able to generate different forces, F(a) and F(c), at the same fiber length $L_{0}$ (*green arrow*). Extrinsic signals such as pH [10], glucagon [11,12], and most prominently, β-adrenergic signals can increase contractility at a given fiber length. These signals operate in the intact animal to regulate cardiac output. But studies in isolated hearts [13], muscle strips [14,15], and single isolated myocytes [16,17] show that heart muscle cells have the *intrinsic* ability to generate different forces at a fixed fiber length. We call this ability the “*intrinsic load-adaptation*” of cardiomyocytes.

Intrinsic load-adaptation is paradoxical. How can a higher contraction force be maintained even when the muscle length has recovered its original length? Because at the steady state length $L_{ss}$ the heart can generate (at least) two different forces, causality requires that the muscle be in different states distinguished by at least two different state variables. According to Starling’s law of the heart one state variable is the muscle fiber length, or more precisely, the fractional change in length or strain. What is the second state variable that enables the myocyte to maintain increased contraction force after the sarcomere length returned to its previous state? What keeps the Anrep mechanism active/activated at ventricular volume that previously generated less force?

We hypothesize that the second state variable is the mechanical stress imposed on the cardiomyocyte. We made this choice because wall stress of the heart normally increases with outflow resistance. What mechanisms enable the myocyte to sense different stresses at the same strain (e.g., points *a* and *c*) and to respond to these different stresses?

**Figure 2:**
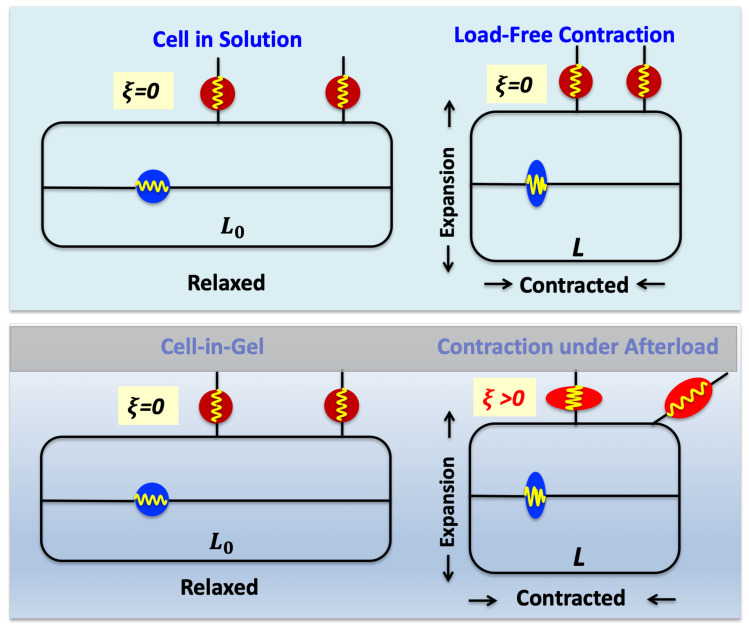
Conceptual ideas underlying the relationship between cell shortening (cell strain ϵ) and surface mechanosensor strain (ξ) during contraction in Tyrode’s solution (**top panel**) or in the gel (**bottom panel**). The red circles and ellipses are the surface mechanosensors; blue circle/ellipse is the internal mechanosensor.

One possible mechanism is having a mechanosensor, conceptualized as a spring, within the myocyte and oriented parallel to the axis of contraction. This mechanosensor is shown as the blue circle in Figure 2. In order for the myocyte to differentiate the stresses at points *a* and *c* in Figure 1 where the fiber lengths are almost identical, the mechanosensor has to be coupled to a high-sensitivity mechanotransducer that can respond to small changes in mechanosensor strain. (This is analogous to the synthetic mechanosensor used in electronic kitchen scales.) This class of mechanosensor is studied another paper from our group (Kazemi-Lari, Shaw, Wineman, Shimkunas, Jian, Hegyi, Izu, and Chen-Izu, in review).

In this paper we focus on a mechanism in which the mechanosensor lies on the surface of the myocyte. This mechanosensor is shown by the red circle/ellipses in Figure 2. We couple the surface mechanosensor model to a simple, yet general, mechano-chemo-transduction (MCT) mechanism.

A key result of this paper is the mathematical proof that autoregulation of contraction amplitude is an inherent property of this coupled mechanosensor—MCT system. This means that within a range of increasing mechanical loading, the system can maintain an approximately constant contraction amplitude. The coupled mechanosensor—MCT model makes some counterintuitive predictions that we confirmed experimentally. The ability of the model to qualitatively predict counterintuitive behavior bolsters our confidence in the framework model for mechanosensing and mechanotransduction.

## 2. Model Description and Results

### 2.1. Conceptual and Mathematical Models

In the heart, the primary force, or stress (force/area) occurs along the longitudinal axis of each myocyte to change the LV volume and eject blood, but secondary 3-dimensional (3-D) stresses also occur. These secondary stresses, involving transverse compression and shear stress, arise for several reasons: (1) the complex shape of the LV, (2) the nonuniform orientation of myocytes across the myocardium wall, (3) blood pressure that creates transverse normal stress at the endocardium, (4) constraint imposed by the pericardium at the epicardium, and (5) other heterogeneities within the myocardium such as the extracellular matrix, coronaries, irregular myocyte shapes, and intermyocyte misalignment. Shearing and transverse stresses can also be generated if adjacent myocytes contract to different extents or at different times.

The Cell-in-Gel system [16] was developed to approximate the stresses experienced by a cardiomyocyte in the working myocardium. In the Cell-in-Gel system, cardiomyocytes are embedded in a viscoelastic gel matrix made of crosslinked polyvinyl alcohol (PVA). The viscous and elastic properties of the gel matrix is tuned by changing the PVA to crosslinker ratio [17]. When the embedded myocyte contracts against the viscoelastic gel, the myocyte experiences 3-D mechanical stresses, namely longitudinal, tensile, transverse compressive, and shear stresses [16,18,19]. As a myocyte contracts along its long axis, it expands in the transverse (perpendicular) directions because the volume of a myocyte is constant. Previous mathematical models for studying the effect of mechanical load on cardiomyocyte have considered only the longitudinal stress acting on the two ends of the cell (e.g., [20,21]). Now we also consider the transverse and shearing stresses acting on surface mechanosensors.

Figure 2 illustrates our hypothesis of how surface mechanosensors (red circles/ellipses) behave when the myocyte contracts in-solution (top panel) vs. in-gel (bottom panel). When the myocyte contracts in solution, the solution presents almost no mechanical resistance so the surface mechanosensors are not deformed as the myocyte expands transversely. However, when the myocyte contracts in situ or in-gel, the viscoelastic gel imposes mechanical resistance that stretches, compresses, and shears the surface mechanosensors. Thus cell-surface mechanosensors are subjected to the stress (force per unit area) from mechanical load.

Figure 2 shows intuitively why the surface mechanosensor strain increases with increasing gel stiffness for any given cell strain. The horizontal axis is the cell strain or fractional shortening defined as $\epsilon =\;\left(L_{0}-L\right)/L_{0}$, where $L_{0}$ is the cell’s resting length and *L* is the length during contraction. Because we are only dealing with contraction our definition of cell strain flips the sign from the usual definition of strain just to avoid the inconvenience of always writing a minus sign. The surface mechanosensor strain ξ is defined in a similar way as the relative change in surface mechanosensor length. Note that while cell strain is routinely measured, the surface mechanosensor strain has not been directly measured experimentally.

**Figure 3:**
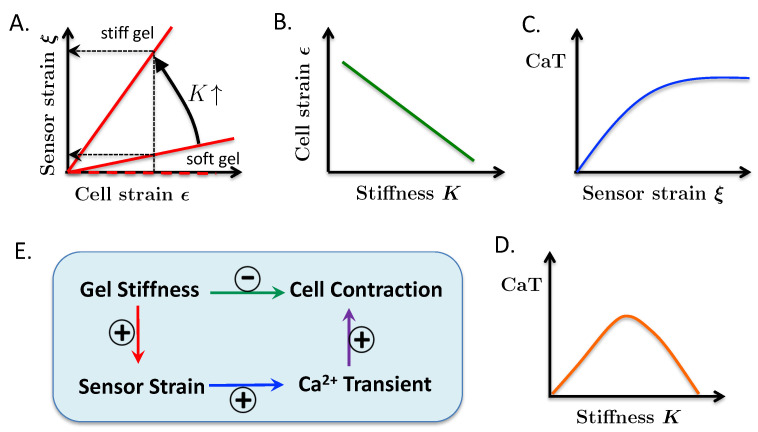
Graphical representation of key ideas of the model. (**A**) Shows that surface mechanosensor strain ξ increases with cell strain ϵ and gel stiffness *K*. In Tyrode’s solution (K=0, *thick, dashed horizontal red line*) the mechanosensor strain is zero regardless of cell strain. (**B**) Shows that at a fixed ${\mathrm{Ca}}^{2+}$ transient (CaT) amplitude, cell strain decreases as the gel gets stiffer. (**C**) Represents our hypothesis that the CaT amplitude increases as the surface mechanosensor strain increases. (**D**) Shows the nonmonotonic relationship between CaT and gel stiffness arising from the reciprocal relationship between cell strain and surface mechanosensor strain with gel stiffness. (**E**) Shows how gel stiffness, sensor strain, and CaT affect cell contraction.

The key ideas of our model are depicted in Figure 3. The relationships between cell strain (ϵ), surface mechanosensor strain (ξ), and gel stiffness (*K*), qualitatively described in Figure 2, are captured in the plots of ξ vs. ϵ in Figure 3A. In the limit of zero gel stiffness—achieved when myocytes contract in Tyrode’s solution—mechanosensor strain is unchanged regardless of cell strain. This case is represented by the dashed red horizontal line. As a larger gel stiffness is used, the ξ–ϵ curve rotates counterclockwise, so for a given cell strain (vertical dashed line) the surface mechanosensor strain will be smaller in the soft gel (lower horizontal dashed line) than in the stiff gel (upper dashed line).

The relationships shown in Figure 3A are expressed in Equation (1) (see below). α(K) is the slope of the ϵ–ξ curve. The zero lower bound on α would occur when the myocyte contracts in Tyrode’s solution. The counterclockwise rotation of the ϵ–ξ line in Figure 3A is assured by the derivative $\alpha^{\prime }\left(K\right)$ being positive. (The prime symbol ${}^{\prime }$ indicates differentiation with respect to the argument in the parentheses.) This inequality means that the surface mechanosensor strain is larger in a stiffer gel than in a softer gel (or Tyrode’s solution) for a given cell strain ϵ.

The idea embodied in Figure 3A is the defining element in our model for explaining the origin of the intrinsic load-adaptation property of cardiomyocytes. The fact that there is no single relationship between cell strain and sensor strain but a continuum of relationships parameterized by the gel stiffness provides a mechanism that enables cardiomyocytes to sense different stresses at the same strain.

To further develop the model we need a relationship between the surface mechanosensor strain, ξ, and the ${\mathrm{Ca}}^{2+}$ transient (**CaT**) amplitude designated in the model by *C*. Based on our previous experimental work [16] on mouse cardiomyocytes and more recent experiments on rabbit cardiomyocytes [17] we propose that *C* is an increasing function of ξ; this relationship is represented by the positive derivative of ϕ(ξ) in (3) and qualitatively in Figure 3C. The upper bound of *M* on ϕ is expected by common sense (${\mathrm{Ca}}^{2+}$ transients must be finite) and is necessary mathematically to keep the system bounded. For our mathematical proofs (see Appendix A) we require that all functions have continuous first derivatives.

To derive a relationship between cell strain and gel stiffness, it is reasonable to assume that, for a given amount of ${\mathrm{Ca}}^{2+}$ release from the sarcoplasmic reticulum, contraction decreases as gel stiffness increases. This assumption is shown qualitatively by the downward slope of the *K*–ϵ line in Figure 3B. Equation (2) defines the relationship between the magnitude of the ${\mathrm{Ca}}^{2+}$ transient, *C*, the gel stiffness, and cell contraction strain ϵ. γ(K) relates the cell strain to CaT. Contraction is determined by the force generated by the myocyte minus the resistive force of the gel. The model does not have a variable for force generation but we assume a monotonic relationship between force generation and *C* based on the works of [22,23].

We require γ(K) to be non-negative so that cell strain increases with increasing CaT. Its derivative with respect to *K* is negative which means that, for a given CaT, the cell contraction is less for greater gel stiffness.

Figure 3E summarizes the interactions between gel stiffness, surface mechanosensor strain, CaT, and cell strain. An increase in gel stiffness increases sensor strain (panel *A*, red arrow) for a given magnitude of cell contraction, which increases the CaT (*C*, blue arrow) that increases contraction (purple arrow). On the other hand, an increase in the gel stiffness decreases cell strain (*B*, green arrow). Thus a change in stiffness has opposing effects that results in a bell-shaped relationship between CaT and gel stiffness (Figure 3D). The Biphasic Theorem (proven in the Appendix A) shows that the bell-shaped relationship between *C* and *K* holds provided the feedback gain (explained below) is large enough.

Equations (1)–(3) comprises our abstract model for mechanosensing in cardiomyocytes.

(1) $$\begin{array}{cc} \xi & =\alpha \left(K\right)\epsilon ,\;\;\alpha \left(K\right)\geq 0\;\mathrm{and}\;\alpha {\left(K\right)}^{\prime }>0 \end{array}$$

(2) $$\begin{array}{cc} \epsilon & =\gamma \left(K\right)C,\;\;\gamma \left(K\right)\geq 0\;\mathrm{and}\;\gamma {\left(K\right)}^{\prime }<0 \end{array}$$

(3) $$\begin{array}{cc} C & =\phi \left(\xi \right),\;\;0<\phi \left(\xi \right)\leq M\;\mathrm{and}\;\phi^{\prime }\left(\xi \right)>0. \end{array}$$

These equations describe how the cardiomyocyte senses mechanical forces (Equation (1)), transduces the forces to chemical signals that control the ${\mathrm{Ca}}^{2+}$ transient amplitude (Equation (3)), and generate contraction (Equation (2)). Accordingly, we call this set of 3 equations the abstract model of mechano-chemo-transduction or MCT model.

*Iterative maps.* Our model for mechanosensing and transduction to a ${\mathrm{Ca}}^{2+}$ signal represented by Equations (1)–(3) is, of course, far too simple to describe the detailed temporal dynamics of ${\mathrm{Ca}}^{2+}$ and contraction. Instead, these equations should be thought of as representing the peak values of each dynamical variable from beat to beat. Despite the simplicity of the model it is rich enough to show the evolution of the mechanosensor strain (ξ), ${\mathrm{Ca}}^{2+}$ transient (*C*), and cell strain (ϵ) by iterative maps of Equation (1).

Assume that the initial (labeled with subscript 0) contraction is $\epsilon_{0}$ then the sensor strain, CaT, and next contraction will be (subscript 1)

(4) $$\xi_{1}=\alpha \epsilon_{0}\;,\;C_{1}=\phi \left(\xi_{1}\right)=\phi \left(\alpha \epsilon_{0}\right)\equiv \phi_{0}\;,\;\epsilon_{1}=\gamma C_{1}=\gamma \phi_{0}.$$

Because it is understood that α and γ depend on a fixed *K*, we drop *K* from the notation. The next iteration gives

(5) $$\xi_{2}=\alpha \epsilon_{1}=\alpha \gamma \phi_{0}\;,\;C_{2}=\phi \left(\xi_{2}\right)=\phi \left(\alpha \gamma \phi_{0}\right)\;,\;\epsilon_{2}=\gamma C_{2}=\gamma \phi \left[\phi \left(\alpha \gamma \phi_{0}\right)\right]$$

The quantity αγϕ(·) recurs often so define Φ=αγϕ and $\Phi_{0}=\alpha \gamma \phi_{0}$. In general, the variables on the *n*-th iteration are

(6) $$\begin{array}{cc} \xi_{n} & =\underset{n-2}{\underset{︸}{\Phi \left(\Phi \left(\cdots \Phi (\Phi_{0}\right)\right)}}\equiv \Phi^{n-2}\left(\Phi_{0}\right) \end{array}$$

(7) $$\begin{array}{cc} C_{n} & =\phi \left(\underset{n-2}{\underset{︸}{\Phi (\Phi \left(\cdots \Phi (\Phi_{0}\right)}}\right)=\phi \left(\Phi^{n-2}\left(\Phi_{0}\right)\right) \end{array}$$

(8) $$\begin{array}{cc} \epsilon_{n} & =\gamma \left(K\right)\phi \left(\underset{n-2}{\underset{︸}{\Phi \left(\Phi \left(\cdots \Phi (\Phi_{0}\right)\right)}}\right)=\gamma \left(K\right)\phi \left(\Phi^{n-2}\left(\Phi_{0}\right)\right) \end{array}$$

**Figure 4:**
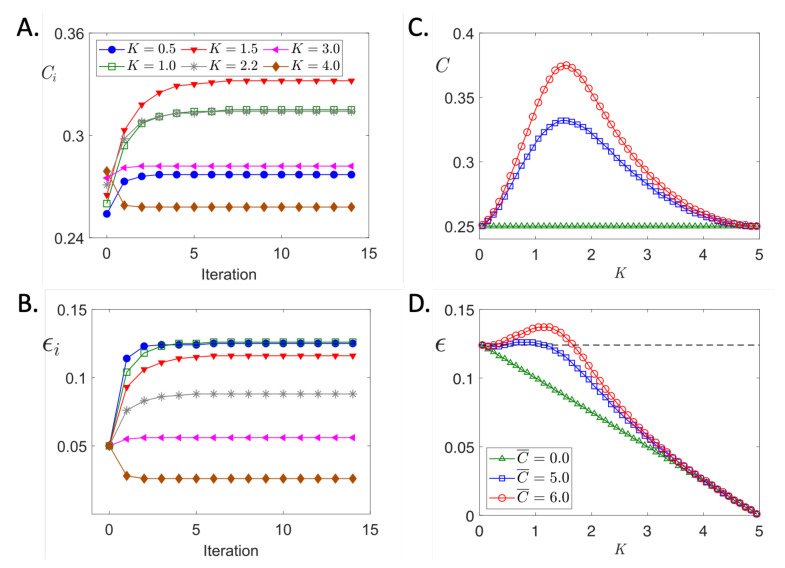
Predicted Ca${}^{2+}$ transient amplitude and cell strain dependence on gel stiffness. (**A**) Shows the time evolution of CaT for values of *K* indicated in the legend. (**B**) Time evolution of cell strain starting at a value of $\epsilon_{0}=0.05$. Curve labels as in (**A**). (**C**) Gel stiffness dependence of steady state values of CaT for different MCT amplifications set by values of $\bar{C}$ given in the legend of panel (**D**). (**D**) Similar to panel (**C**) but cell strains are shown. Simulation parameters: $K_{\infty }=5\;,\;K_{c}=0.5\;,\;K_{g}=1.2$, $\epsilon_{0}=0.05\;,\;\bar{C}=5$ (except as noted), δ=0.1, $\alpha_{0}=1\;,\;n_{c}=2\;,\;C_{0}=0.25$.

Boundedness on ϕ (Equation (3)) ensures boundedness on $\xi_{n}$, $C_{n}$, and $\epsilon_{n}$. Iterative evolution of *C* and ϵ are shown in Figure 4A,B.

*The usefulness of writing the model in an abstract form.* Equations (1)–(3) comprise an abstract model in the sense that we do not specify the form of α, γ, and ϕ except for boundedness conditions and signs of derivatives. The value of such an abstract representation is that the derived results are not wedded to any particular representation of these three functions out of an infinitude of possibilities. In the Appendix A we show that all functions that satisfy the conditions of Equations (1)–(3) have the following properties: (*a*) Iterative maps of these functions always converge and the convergence is monotonic. (*b*) An increase in contraction always produces an increase in CaT that, in turn, causes an increase in contraction. In other words, the system represented by Equations (1)–(3) is regenerative but, because of the convergence property, is stable. (*c*) The response of the system is not instantaneous like the Frank-Starling mechanism. Instead, like the Anrep effect, the system takes time to reach a steady state. (*d*) Autoregulation of contractility occurs provided $\phi^{\prime }\left(\xi \right)$ is large enough.

### 2.2. Further Insights from a Specific Example

The results in the Appendix A are universal but abstruse. Further insights come by choosing specific functional representations of α, γ, and ϕ that satisfy the conditions of Equations (1)–(3). Equations (9)–(11) comprise one such representation. We chose these forms because they satisfy the conditions of Equations (1)–(3) and because they are simple and intuitively reasonable.

(9) $$\begin{array}{cc} \alpha (K) & =\alpha_{0}\frac{K}{K_{g}+K},\;\alpha_{0}\geq 0 \end{array}$$

(10) $$\begin{array}{cc} \gamma (K) & =\delta \cdot (K_{\infty }-K) \end{array}$$

(11) $$\begin{array}{cc} \phi (\xi ) & =C_{0}+\frac{\bar{C}\xi^{n_{c}}}{K_{c}^{n_{c}}+\xi^{n_{c}}},\;\;n_{c}>0 \end{array}$$

We used these equations in the iterative map, Equations (6)–(8). Figure 4A,B show the evolution of CaT and cell strain, respectively, for selected values of gel stiffness *K* ranging from very soft (K=0.5) to very stiff (K=4). As predicted by Theorem A1 in the Appendix A, $C_{i}$ and $\epsilon_{i}$ evolve monotonically to steady state values for a given *K*. An important feature shown by these curves is that the steady state values of *C* first increase then decrease as gel stiffness increases. For example, the steady state value of *C* is about 0.27 for K=0.5 (blue circles) but increase to about 0.31 when *K* is increased to 1.0 (green open squares). This increase in CaT is sufficient to maintain the contraction amplitude ϵ (Figure 4B) the same despite the greater mechanical load the cell must work against. But a further stiffening of the gel to K=3 (magenta left-triangles) results in the decrease of *C* to about 0.27 and ϵ to about 0.05. When *K* is 1.0 (green open squares) or 2.2 (gray asterisks), the steady state CaT happen to be about the same (the two *C* curves overlap) but the strain curves are clearly separated. This makes sense because for the same CaT, the cell will contract less in a stiffer environment.

The biphasic behavior of the steady state *C* and ϵ values are more clearly seen in the summary plot of Figure 4C,D. The prediction that a cell will contract at a constant amplitude despite a stiffer gel seemed counterintuitive.

### 2.3. Experimental Test of Biphasic Prediction

**Figure 5:**
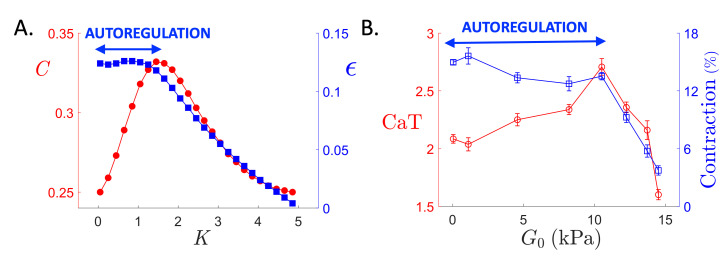
Comparison of model predictions and experimental measurements. (**A**) consolidates the steady state CaT (red circles) and cell strain (blue squares) dependence on stiffness *K* from Figure 4C,D ($\bar{C}=5.0$). (**B**) shows the dependencies of the steady state CaT indexed by the fura-2 ratio (red open circles) and steady state contraction amplitude measured by the fractional shortening (blue open squares) on the instantaneous shear modulus $G_{0}$. We say that autoregulation (blue double-headed arrow) occurs in the range of *K* or $G_{0}$ where cell strain is approximately constant.

To test the model prediction we generated gels with varying viscoelastic properties by changing the ratio of boronate crosslinker to PVA. For each crosslinker—PVA combination we measured the storage $G^{\prime }$ (∼elasticity) and loss $G^{\prime \prime }$ (∼viscosity) moduli from which the instantaneous elastic shear modulus $G_{0}$ was calculated [17] and Kazemi-Lari et al. [24]. Cardiomyocytes from the left ventricle of rabbits were embedded in gels with varying crosslinker concentrations and electrically stimulated to contract. Fura-2 fluorescence ratio (a measure of CaT) and fractional shortening were measured as described in our experimental paper [16]. The experimental results shown in Figure 5B are taken from our paper [17], which provide the experimental protocols.

These experimental data bear striking similarity to the model predictions in Figure 5A. First, notice that in both the model and experiments, as the gel stiffens (increasing *K* and $G_{0}$), CaT (red circles) initially rises then falls. Second, for a range of gel stiffness, myocyte contraction (blue squares) remains relatively constant (blue double-headed arrows) despite the increased mechanical loading. The existence of this region, called the autoregulatory zone, is guaranteed by Theorem A2 (Appendix A). Third, as the gel stiffness increases beyond this autoregulatory zone (∼10 kPa) both CaT and fractional shortening decrease precipitously.

It is important to keep in mind that the experiments (Figure 5B) were done *after* the counterintuitive modeling predictions were made. The striking similarity between the predicted and measured CaT and cell contraction curves gives us confidence that our conceptual idea for how mechanical load is being sensed by the cardiomyocyte (Figure 2) is fundamentally sound.

## 3. Discussion

### 3.1. Experimental Underpinnings for the MCT Model

We developed the model in this paper to account for two contrasting sets of results from our experiments done in the Cell-in-Gel system and experiments by others done with different systems. The first contrasting set of observations is in the change of the CaT amplitude when cardiomyocytes contract auxotonically (contraction under changing load) compared to unloaded contraction. We found that the CaT amplitude is larger when cardiomyocytes contract while embedded in a viscoelastic gel compared to the CaT amplitude when contracting in Tyrode’s solution, which offers little mechanical resistance to contraction [16,17]. By contrast, White et al. [25] found no change in the CaT amplitude in going from load-free to auxotonic contraction. In their system, cardiomyocytes contracted against a load imposed by a flexible carbon fiber attached to one end of the long axis of the cell while the other end remained fixed with a stiff carbon fiber. Importantly, the cells were immersed in Tyrode’s solution that offers little mechanical resistance.

The second set of contrasting results come from experiments that inhibit nitric oxide (NO) synthase (NOS). We found that the frequency of ${\mathrm{Ca}}^{2+}$ sparks during diastole increased when cardiomyocytes contracted in the viscoelastic gel versus Tyrode’s solution and inhibition of NOS by N(*ω*)-nitro-l-arginine methyl ester (L-NAME) or the specific NOS1 inhibitor N(*ω*)-propyl-l-arginine hydrochloride (L-NPA) reduced the spark frequency to that seen when cells contract in Tyrode’s solution. By contrast, Prosser et al. [26] found that L-NAME had no effect on ${\mathrm{Ca}}^{2+}$ spark frequency in unstretched or stretched cardiomyocytes. In their system cardiomyocytes were stretched with glass fibers attached to one end of the long axis of the cell and in the middle of the cell. As with White et al. in their system the cells were immersed in Tyrode’s solution that, as mentioned, imposes little mechanical force on the cardiomyocyte.

We suggested that the salient difference between our experiments and those of others lay in the so-called dimensionality of forces [19], that is the number of dimensions against which the cardiomyocytes did external work against. In the Cell-in-Gel system the dimension is 3 while in the systems of White et al. and Prosser et al. the dimension is 1. The difference in dimensionalities suggested to us that surface mechanosensors that lie on the lateral surface of the cardiomyocytes were being activated by transverse and shear forces during contraction in the gel but not in Tyrode’s solution [19,27].

### 3.2. Biphasic CaT Response and Autoregulation in the MCT Model

The MCT model given in abstract form by Equations (1)–(3) or in the specific form by Equations (9)–(11) are mathematical translations of the intuitive ideas presented in [19,27] and Figure 2. Equations (9)–(11) made two predictions that defied our intuition. The *biphasic response* prediction is that the CaT amplitude would rise then fall as the gel stiffens as shown in Figure 4C. The *autoregulation* prediction, even more surprising than the first, is that fractional shortening would remain constant or even increase despite an increase in gel stiffness, at least up to a point as shown in Figure 4D.

We tested these predictions experimentally by varying the gel’s viscoelastic properties and measuring the steady state CaT amplitude and fractional shortening. Experimental matches of complex qualitative features (biphasic response, autoregulation) are stringent tests of the model. Thus the experimental results would clearly either debunk or validate the model. The concordance between the experimental and model predictions shown in Figure 5 supports the idea that the model for MCT in cardiomyocytes given by Equations (9)–(11) is broadly correct.

### 3.3. Stability, Biphasic CaT Response, and Autoregulation Are Natural Emergent Properties of MCT

Although the model results shown in Figure 4 and Figure 5 are from one specific model, given by Equations (9)–(11) and a few sets of parameters, the results are general because these equations satisfy the conditions of the abstract model equations given by Equations (1)–(3). Therefore Theorems A1–A3, proven in the Appendix A, guarantees stability and convergence, biphasic CaT response, and autoregulation.

**Figure 6:**
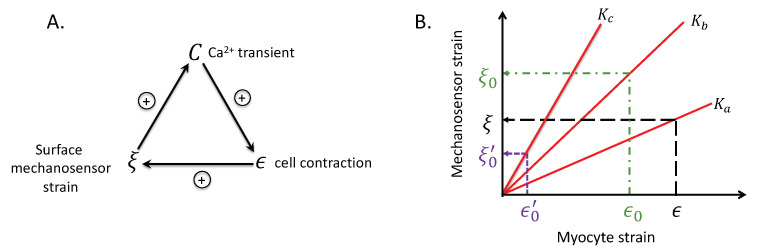
Positive feedback is inherent in the model. (**A**) Closed loop structure of model. (**B**) Effect of loop structure on myocyte and mechanosensor strains. ξ = surface mechanosensor strain, ϵ = cell strain, *C*, ${\mathrm{Ca}}^{2+}$ transient amplitude, and *K*, gel stiffness.

Theorems are more than proven mathematical assertions. In the context of this paper, their universality implies that autoregulation, biphasic CaT, and stability naturally emerge from the structure of interactions between the surface mechanosensor strain (ξ), cell strain (ϵ), and the CaT (*C*). This structure of interactions, given by Equations (1)–(3), is depicted in Figure 6A.

### 3.4. What the Theorems Tell Us about MCT

Figure 6A shows that the interaction of *C*, ξ, and ϵ have a closed loop structure. The positive signs indicate that the variable at the tail of the arrow enhances the variable at the tip of the arrow. Because each variable enhances each other we might expect explosive growth but Theorem A1, the Convergence theorem, guarantees convergence. Convergence of the CaT and cell strain are shown in Figure 4A,B. (We do not show the convergence of the surface mechanosensor strain ξ because this variable is not measured in experiments). What limits explosive growth is the bound *M* on the ${\mathrm{Ca}}^{2+}$ transient given by ϕ in Equation (3).

By autoregulation of contractility we mean that the change in the contraction amplitude ϵ as the gel stiffness *K* changes is nil. The Autoregulation theorem states that for a range of stiffness, the contraction amplitude will not change much, provided the MCT feedback gain dϕ/dξ is large enough. The flatness of the ϵ–*K* curve (blue squares) for a range of *K* in Figure 4D illustrate the meaning of the Autoregulation theorem.

The Biphasic Theorem states that the CaT *C*, the cell strain ϵ, and the mechanosensor strain ξ (that we cannot currently measure) can be nonmonotonic functions of gel stiffness *K*. The nonmonotonic (hump) behavior is shown in Figure 4C,D. Note that nonmonotonicity is not necessary; setting the feedback gain dϕ/dξ to zero eliminates the hump as shown by green triangle curves.

In particular, we can use the model shown in Figure 3, which conform to Equations (1)–(3), to illustrate intuitively how autoregulation and the biphasic behavior arise.

Suppose that the gel has stiffness $K_{a}$ and the myocyte has a steady state contraction amplitude of ϵ shown in Figure 6B (black, long-dashed lines). The surface mechanosensor has strain ξ. Now imagine that the stiffness of the gel suddenly increases to $K_{b}$. Let the first contraction after the stiffening have magnitude $\epsilon_{0}$. We know that $\epsilon_{0}$ must be less than the previous one by common sense and by Equation (2). Figure 6B (green, dash-dot lines) shows that despite the smaller cell contraction the mechanosensor strain $\xi_{0}$ is larger than ξ because the slope $K_{b}>K_{a}$. The loop structure in Figure 6A shows that the larger $\xi_{0}$ will result in a larger CaT that will, in turn, lead to a larger cell contraction and a larger ξ, a larger *C*, and a larger cell contraction, and so on. Runaway growth is precluded because the CaT is bounded by *M*. The actual values of $C_{1}$, $\xi_{1}$, and $\epsilon_{1}$ are determined by Equation (4).

The case just described explains the rising phase of the ${\mathrm{Ca}}^{2+}$ transient–stiffness curves in Figure 4C,D. The falling phase can be explained similarly. Suppose instead that the gel stiffness increases from $K_{a}$ to $K_{c}$ in Figure 6B (purple, short-dashed lines). Now the stiffness is so great that the first contraction following the stiffening is the much smaller $\epsilon_{0}^{\prime }$. This causes the mechanosensor strain $\xi_{0}^{\prime }$ to be smaller than ξ so consequently the CaT will be smaller so the next myocyte contraction amplitude $\epsilon_{1}^{\prime }$ will be even smaller. This downward spiral accounts for the falling phase of the biphasic *C* and ϵ curves.

### 3.5. Intrinsic Inotropy

The observation that the single cardiomyocyte isolated from external neurohormonal signals can increase the amplitude of its CaT in stiffer gels shows cardiomyocytes possess an intrinsic ability to increase inotropy. This ability appears to depend on the surface mechanosensors that detect transverse and shear forces because White et al. [25] found no change in the CaT amplitude when cardiomyocytes contracted auxotonically in Tyrode’s solution. Further evidence for the involvement of surface mechanosensors come from O. Cingolani et al. [5] who, as White et al., used the carbon fiber technique and found only a modest 10% increase of the CaT amplitude in mouse cardiomyocytes. By contrast we found CaT increase of 34% in mouse [16] and 76% in rabbit [17].

### 3.6. Intrinsic Inotropy and the Anrep Effect

The Anrep effect describes the increase in contractility of the heart in response to an increase in afterload. An important mediator of the Anrep effect is β-adrenergic stimulation as Anrep himself found [4]. Other extrinsic factors such as pH [10], glucagon [11,12,28], and angiotensin [29,30] are also likely to be involved because when β-adrenergic receptors are blocked [28,31], saturated [14], or when they are reduced [8] the Anrep effect still occurs.

The intrinsic inotropy that the Cell-in-Gel experiments revealed adds a new dimension to our understanding of the cellular basis of the Anrep effect. In these experiments the gel resists myocyte contraction simulating the mechanical environment of the myocyte in the working myocardium as wall stress increases.

Myocytes contracting in-gel start from the same slack length of 1.8–2.0 μm without prestretch, so the effect of the Frank-Starling mechanism is constant. Thus the intrinsic inotropy can contribute to the variable force production at the same muscle length shown in Figure 1. The mechanism underlying this variable force production is the change in surface mechanosensor strain for the same cell strain depending on gel stiffness shown in Figure 3 and Figure 6.

### 3.7. Strength—Limitation Duality of the Model

Writing the mathematical representation of the MCT model with 3 abstract (Equations (1)–(3)) or concrete (Equation (9)–(11)) equations is both a strength and a limitation. An important limitation of this approach is that specific signaling pathways are not identified. The mathematical model only requires that mechanosensor strain and the CaT are non-negatively related and bounded. The model is completely silent on the origins of this relationship.

Our experiments point to the critical role of NO signaling in the autoregulation of contractility under mechanical loading in the Cell-in-Gel system [16,17]. Our working hypothesis is that the dystrophin-glycoprotein complex (DGC) is functioning as the surface mechanosensor. DGC has both components that lie on the cell surface (dystroglycans) and within the myocyte (dystrophin) making this macromolecular complex suitable as a mechanosensor [32,33]. Furthermore, NOS1 is linked to dystrophin [34,35] so we envision mechanical forces transmitted via dystrophin to NOS1 modulating NOS1’s activity. Experiments are underway in our lab to test this hypothesis.

We also found that ${\mathrm{Ca}}^{2+}$/calmodulin-dependent protein kinase (CaMKII) is involved in load-mediated ${\mathrm{Ca}}^{2+}$ regulation [16]. Our working hypothesis is that a change in surface mechanosensor strain is coupled to activation of NOS1, which in turn modulates CaMKII [36,37]. CaMKII, depending on level of activation and compartmentalization has variable effects on ryanodine receptors [36,38,39,40] and SERCA${}_{2A}$[38,39,40] that can either increase or decrease the CaT and fractional shortening.

The simplicity of the model is also a strength. The model comprises just 3 interacting parts and makes counterintuitive predictions that experiments confirmed. Furthermore, the constraints on the model are mild, consisting of just signs of the derivatives and bounds. The surprising conclusion we can draw is that a simple and robust mechanism described in this conceptual model is sufficient to explain autoregulation of contraction in cardiomyocytes.

## Data Availability

Not applicable.

## Abbreviations

The following abbreviations are used in this manuscript:

CaT	${\mathrm{Ca}}^{2+}$ transient
EDV	end-diastolic volume
F-S	Frank-Starling
MCT	mechano-chemo-transduction
L-NAME	N(*ω*)-nitro-l-arginine methyl ester
L-NPA	N(*ω*)-propyl-L-arginine hydrochloride
LV	left ventricle or left ventricular
NO	nitric oxide
NOS	nitric oxide synthase

## Appendix A

### Appendix A.1. Monotonic Convergence of Equations (1)–(3)

Let ξ, ϕ, and ϵ be given by Equations (1)–(3) in the main text. We prove that the iterative map always converges monotonically.

**Theorem** **A1** (Convergence). *If $\phi_{k+1}>\phi_{k}$ then $\phi_{k+2}>\phi_{k+1}$ and $\left\{\phi_{k}\right\}$ is a monotonically increasing convergent sequence.*

**Proof of Theorem A1** By the assumption of the theorem $C_{k+1}=\phi_{k+1}>\phi_{k}=C_{k}$ and this implies that $\epsilon_{k+1}>\epsilon_{k}$ from (2). Therefore, $\xi_{k+2}=\alpha \epsilon_{k+1}>\alpha \epsilon_{k}=\xi_{k+1}$. Because $\phi^{\prime }\left(\xi \right)>0$ it immediately follows that $\phi_{k+2}=\phi \left(\xi_{k+2}\right)>\phi \left(\xi_{k+1}\right)=\phi_{k+1}$. This completes the proof of monotonicity. Convergence follows naturally because ϕ is bounded by *M* so by the Monotone Convergence Theorem the sequence $\phi_{k}$ converges. □

Note that if $\phi_{k+1}<\phi_{k}$ a similar argument shows that $\left\{\phi_{k}\right\}$ is a monotonically decreasing convergent sequence.

There are two immediate corollaries. The first is that $\epsilon_{k+2}>\epsilon_{k+1}$ so contraction amplitude increases monotonically and converges. The second is that an increase in contraction always produces an increase in ${\mathrm{Ca}}^{2+}$ that, in turn, causes an increase in contraction. This feedback loop, shown in Figure 6, is bounded despite it being regenerative.

### Appendix A.2. Autoregulation Occurs Given Sufficient MCT Amplification

**Theorem** **A2** (Autoregulation). *By autoregulation we mean that*

(A1) $$\epsilon^{\prime }\left(K\right)=\frac{d\epsilon }{dK}\approx 0.$$

*Equality of $\epsilon^{\prime }\left(K\right)$ to zero can always be obtained provided $\phi_{\xi }\left(\xi \right)$ is large enough.*

**Proof of Theorem A2** From (3) and (1) we get

(A2) $$C=\phi \left(\xi (K)\right)=\phi \left(\alpha (K)\epsilon \right).$$

Therefore from (2) we get

(A3) $$\epsilon =\gamma \left(K\right)\phi \left(\alpha (K)\epsilon \right).$$

Then

(A4) $$\epsilon^{\prime }\left(K\right)=\frac{d\gamma }{dK}\left(K\right)\;\phi \left(\alpha (K)\epsilon \right)+\gamma \left(K\right)\frac{d\phi }{dK}.$$

Applying the chain rule gives

(A5) $$\frac{d\phi }{dK}=\frac{d\phi }{d\xi }\left(\alpha (K)\epsilon \right)\cdot \frac{d\alpha }{dK}\cdot \epsilon .$$

Substituting (A5) into (A4) gives

(A6) $$\frac{d\epsilon }{dK}=\underset{<0}{\underset{︸}{\gamma_{K}\left(K\right)}}\underset{>0}{\underset{︸}{\phi \left(\alpha (K)\epsilon \right)}}+\underset{>0}{\underset{︸}{\gamma (K)}}\underset{>0}{\underset{︸}{\phi_{\xi }\left(\alpha (K)\epsilon \right)}}\underset{>0}{\underset{︸}{\alpha_{K}}}\underset{>0}{\underset{︸}{\epsilon }}.$$

The subscript on the function (e.g., $\phi_{\xi }$) indicates differentiation with respect to the subscripted variable. The sign of each term on the right hand side of (A6) is given in (1)–(3). Because the first and second products have different signs, it is always possible to set $\epsilon^{\prime }\left(K\right)=0$ provided that the MCT amplification, defined as

(A7) $$\phi_{\xi }\left(\alpha (K)\epsilon \right)$$

is large enough to compensate for $\gamma_{K}$. We assume that all functions in (1)–(3) are (at least) once differentiable so by continuity there is a neighborhood about $K^{*}$, where $\epsilon^{\prime }\left(K^{*}\right)=0$, in which $\epsilon^{\prime }\left(K\right)\approx 0$. □

### Appendix A.3. Biphasic C(K), ϵ(K), and ξ(K) Curves

Figure 4 shows that the plots of C(K) and ϵ(K) can be non-monotonic. Here we show that this non-monotonic behavior arises from the structure of the model given by (1)–(3).

**Theorem** **A3** (Biphasic). *The steady state values of ξ, C, and ϵ can have a non-monotonic (biphasic) dependence on K.*

**Proof of Theorem A3** To do this we show that the derivatives of ξ, *C*, and ϵ with respect to *K* can be zero. Application of the product rule and chain rule gives

(A8) $$\xi_{K}=C\left[\underset{>0}{\underset{︸}{\alpha_{K}}}\underset{>0}{\underset{︸}{\gamma }}+\underset{>0}{\underset{︸}{\alpha }}\underset{<0}{\underset{︸}{\gamma_{K}}}\right]=C\frac{\partial }{\partial K}\left(\alpha \gamma \right)$$

The signs in (A8) are from (1)–(3). Because the two terms in the sum have opposite signs, $\xi_{K}$ can be zero. Similarly,

(A9) $$C_{K}=\phi_{\xi }\xi_{K}.$$

This shows that when $\xi_{K}=0$, so is $C_{K}$. Finally, Equation (A6) shows that $\epsilon_{K}$ can be zero. Because ξ, *C*, and ϵ are positive, the extrema are maxima. □

Note that we have shown that ξ, *C*, and ϵ can have a biphasic response to *K*. Theorem A3 does not state that the biphasic response is necessary. For example, setting $\phi_{\xi }\equiv 0$ results in ϵ(K) decreasing linearly as shown by the green-triangle line in Figure 4D.
